# Expending Role of Microsatellite Instability in Diagnosis and Treatment of Colorectal Cancers

**DOI:** 10.1007/s12029-017-9991-0

**Published:** 2017-07-11

**Authors:** Liisa Chang, Minna Chang, Hanna M. Chang, Fuju Chang

**Affiliations:** 1 Department of Medicine, Medway NHS Foundation Trust, Gillingham, Kent UK; 2 Faculty of Medicine, Imperial College London, South Kensington Campus, London UK; 3 Division of Cancer Studies, King’s College London, London, UK; 4 Department of Histopathology, Guy’s & St Thomas’ Hospitals NHS Foundation Trust, Westminster Bridge Road, London, SE1 7EH UK

**Keywords:** Microsatellite instability, Mismatch repair, Lynch syndrome, Colorectal cancer, Cancer treatment, Chemotherapy, Immunotherapy

## Abstract

**Background:**

Colorectal carcinomas with high-frequency microsatellite instability (MSI-H) account for 15% of all colorectal cancers, including 12% of sporadic cases and 3% of cancers associated with Lynch syndrome (also known as hereditary nonpolyposis colorectal cancer syndrome, HNPCC). Lynch syndrome is an autosomal dominant hereditary cancer syndrome, caused by germline mutations in mismatch repair genes, including MLH1, MSH2, MSH6 and PMS2.

**Methods:**

Published articles from peer-reviewed journals were obtained from PubMed, Google Scholar and Clinicaltrials.gov. Based on the recent research data, we provide an update on the MSI testing, along with the evolving role of MSI in diagnosis, prognosis and treatment of colorectal cancers.

**Results:**

Studies have led to significant advances in the molecular pathogenesis and clinicopathological characteristics of MSI-H colorectal cancers. Emerging evidence suggests that colorectal cancers with MSI-H show different outcome and treatment response from those with microsatellite stable (MSS) tumors. Therefore, MSI testing is essential not only in the genetic context, but it may also have important prognostic and predictive value of response to chemotherapy and immunotherapy.

**Conclusions:**

Many experts and professional authorities have recommended a universal MSI testing in all individuals newly diagnosed with colorectal cancers.

## Introduction

Colorectal cancer is a major health problem in Western countries, representing the second most commonly diagnosed malignancy in males and third in females and accounting for about 700,000 deaths per year [1, 2]. The majority of colorectal cancers display chromosomal instability and follow the classical adenoma-carcinoma progressive pathway. However, a subset of 15% of colorectal cancers displays DNA mismatch repair (MMR) deficiency and shows high-level microsatellite instability (MSI-H) [2, 3]. Colorectal cancers with MSI-H can occur as sporadic fashion or in the context of Lynch syndrome, also known as hereditary non-polyposis colorectal cancer syndrome (HNPCC) [4, 5]. Thus, colorectal cancers can be classified under two molecular phenotypes, i.e., microsatellite stable (MSS) and microsatellite unstable or MSI-H phenotype [6].

MSI-H colorectal cancer has distinct clinicopathological features, including younger age of onset, proximal location, florid lymphocytic reaction, mucinous/signet ring differentiation and medullary growth pattern [6–8]. MSI is a critical DNA marker for the diagnosis of Lynch syndrome. Recent data also suggest that MSI status in colorectal cancers could provide valuable information for prognostic estimation and treatment stratification [9–11]. We provide here a brief review on recent development of MSI in diagnosis, prognosis and treatment of colorectal carcinoma.

## Microsatellite Instability Testing

Lynch syndrome is caused by inherited defects in mismatch repair (MMR) genes MLH1, MSH2, MSH6 and PMS2 [4–6]. The presence of MSI-H and/or the absence of one or more of the MMR proteins by immunohistochemistry (IHC) in the tumor suggest MMR deficiency. MSI-H can be also caused by somatic hypermethylation of the MLH1 promoter, which is often associated with a BRAF c.1799T>A (p.V600E) mutation [12–14]. These somatic mutations are typically associated with sporadic colorectal carcinomas. MSI can be tested by either polymerase chain reaction (PCR)-based DNA technique or immunohistochemical staining on tumor tissue.

### Steps for the Microsatellite Instability DNA Testing

MSI DNA testing is a PCR-based method that amplifies DNA at several microsatellite sites from a person’s tumor tissue sample. Interpretation of the profiles requires a comparison with normal DNA from each patient. The National Cancer Institute Workshop agreed on five microsatellite markers necessary to determine MSI that include two mononucleotide markers—BAT25/26 and three dinucleotide markers—D2S123, D5S346 and D17S250 [15, 16]. Some laboratories use commercially available testing kit, such as five-marker mononucleotide or quasimonomorphic panel [17, 18]. All of these markers are highly concordant with respect to the testing results [16]. Testing steps by PCR technique are listed in Table 1.

**Table 1:** Steps for the microsatellite instability DNA testing

(1) Do a microsatellite instability test.
(2) If the microsatellite instability test result is positive, use sequential BRAF V600E and MLH1 promoter hypermethylation testing to differentiate sporadic and Lynch syndrome-associated colorectal cancers. First do a BRAF V600E test.
(3) If the BRAF V600E test is negative, do an MLH1 promoter hypermethylation test.
(4) If the MLH1 promoter hypermethylation test is negative, confirm Lynch syndrome by genetic testing of germline DNA.

Based on the MSI status, colorectal cancers can be classified into three groups: (1) if 30% or more of the repeats are unstable, a tumor is classified as MSI-high (MSI-H); (2) if fewer than 30% of repeats are unstable, a tumor is classified as MSI-low (MSI-L); and (3) if no repeats are unstable, a tumor is classified as microsatellite stable (MSS) [16]. A MSI-L profile does not appear to be a good predictor of Lynch syndrome, so this result is grouped with the MSS type and does not lead to further testing.

### Steps for the Immunohistochemistry Testing

Immunohistochemistry is widely used to identify the loss of one or more of the mismatch repair proteins (MLH1, MSH2, MSH6 and PMS2) [6, 8, 16]. These MMR proteins are usually expressed in normal tissue and show positive nuclear staining on IHC. The absence of specific staining suggests an underlying inactivation of one or more MMR genes. Tumors displaying loss of an MMR protein can be collectively referred to as MMR deficiency and are considered to be MSI-H, whereas those with intact MMR proteins are expected to be MSS or MSI-low [6, 8, 16].

**Fig. 1:**
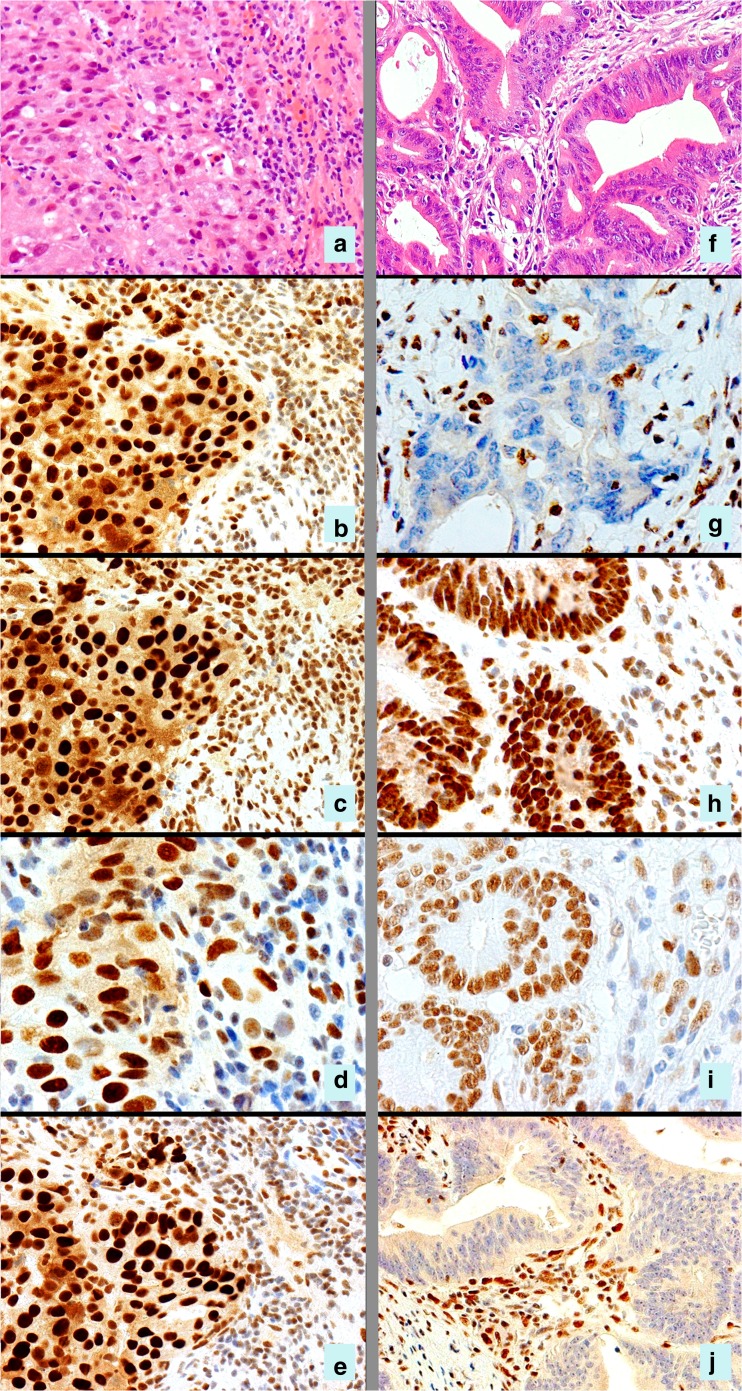
Immunohistochemistry for mismatch repair proteins in two colonic cancers. Patient 1 (panels **a**–**e**) shows a poorly differentiated adenocarcinoma (**a**) which is positive for MLH1 (**b**), MSH2 (**c**), MSH6 (**d**) and PMS2 (**e**), indicating MSS phenotype. Patient 2 (panels **f**–**j**) shows a moderately differentiated adenocarcinoma (**f**) with a MSI-H phenotype. Note the loss of nuclear staining for MLH1 (**g**) and PMS2 (**j**) but normal staining for MSH2 (**h**) and MSH6 (**i**). This tumor was subsequently tested for BRAF V600E and MLH1 promoter hypermethylation and did not reveal any abnormality. The findings in patient 2 are suggestive of Lynch syndrome; therefore, DNA germline testing is recommended

The most common abnormal IHC staining pattern is simultaneous loss of MLH1 and PMS2, with normal staining of MSH2 and MSH6 (Fig. 1). This could indicate either Lynch syndrome or MMR deficiency in a sporadic tumor. Further testing for BRAF V600E mutation and MLH1 promoter hypermethylation can differentiate sporadic tumor from Lynch syndrome-associated cancer [6, 16]. Testing steps by immunohistochemical staining are listed in Table 2. The other IHC profiles, such as combined MSH2/MSH6 loss or isolated loss of MSH6 or PMS2, are more likely to be associated with Lynch syndrome due to a germline mutation in one of these genes [6, 16]. Germline mutation analysis can be done on blood leukocyte DNA or normal tissue of the patient.

**Table 2:** Steps for the immunohistochemistry testing

(1) Do an immunohistochemistry 4-panel test for MLH1, MSH2, MSH6 and PMS2.
(2) If the MLH1 immunohistochemistry result is abnormal, use sequential BRAF
(3) V600E and MLH1 promoter hypermethylation testing to differentiate sporadic and Lynch syndrome-associated colorectal cancers. First do a BRAF V600E test.
(4) If the BRAF V600E test is negative, do an MLH1 promoter hypermethylation test.
(5) If the MLH1 promoter hypermethylation test is negative, confirm Lynch syndrome by genetic testing of germline DNA.

In term of steps 2–4, if the MSH2, MSH6 or PMS2 immunohistochemistry results are abnormal, confirm Lynch syndrome by subsequent genetic testing of germline DNA

### Concordance Rate Between MSI and IHC Testing

Both MSI DNA testing and IHC are sensitive and specific. The reported sensitivity of MSI DNA testing is 89% for MLH1/MSH2 and 77% for MSH6 [19]. The reported sensitivity of IHC for the MMR proteins is 77 to 83% [20, 21]. The concordance rate between IHC and MSI testing is over 92% [20, 21]. In practice, PCR and IHC testing often act as complementary tests; while both are sensitive and specific for mismatch repair deficiency, neither is perfect, and both will miss some mismatch repair-deficient tumors. To increase the detection rate, these two tests may be performed synergistically to detect cases that maybe missed by either test alone [22]. Easy performance and cost-effectiveness are two of the advantages of IHC. Moreover, IHC testing is helpful in identifying the specific defective protein and can guide germline testing to that specific gene.

## Diagnostic Role of MSI Testing

### MSI Deficiency and Lynch Syndrome

MSI deficiency is the hallmark of genetic aberration of Lynch syndrome [3–5]. This syndrome is an autosomal dominant disorder, caused by a germline mutation in one of the mismatch repair genes: MLH1, MSH2, MSH6 and PMS2 [3–5, 20–23]. The majority of Lynch patients can be attributed to the mutation of MLH1 or MSH2, accounting for about 90% of the cases identified [23, 24]. The mutation of MSH6 only accounts for a small portion of Lynch syndrome. Isolated loss of PMS2 is rare in patients with Lynch syndrome [23–25].

**Fig. 2:**
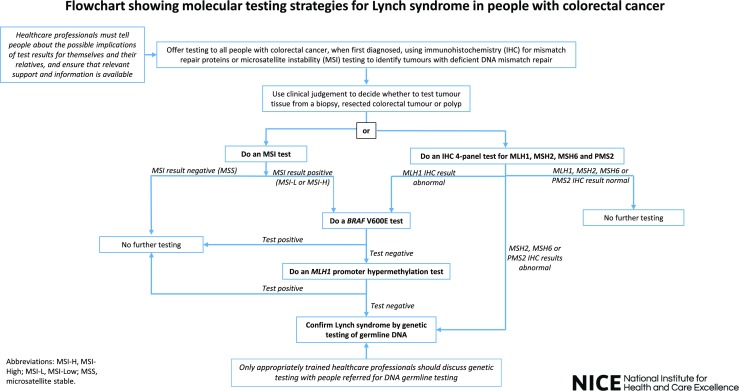
NICE recommended universal screening strategy for Lynch syndrome Reproduced from reference [60]

The flowchart in Fig. 2 shows MSI testing strategies for Lynch syndrome in people with colorectal cancer.

Lynch syndrome accounts for about 3% (one in 30) of colorectal cancers and is estimated to lead to over 1100 colorectal cancers each year in the UK. The diagnosis of Lynch syndrome is essential since the patient has a high risk for developing many other cancers and needs appropriate surveillance. It is also important to identify family members carrying a defect MSI gene because they are at increased risk of developing cancers as well. The lifetime risk of colorectal cancer in individuals with Lynch syndrome is between 30 and 70%, in contrast to 5.5% in the general population [5, 26]. Patients with Lynch syndrome also have a high risk of developing extra-colorectal cancers, including endometrial carcinoma, ovary, small bowel, stomach, bladder, brain, kidney, biliary tract and gallbladder cancers, and skin sebaceous tumors [27–29]. For endometrial cancer, the cumulative risk in Lynch syndrome patients by 70 years is between 32 and 42% [27, 28].

Patients with Lynch syndrome have earlier age of colorectal cancer diagnosis at 44–61 years compared with 69 years in sporadic cases [26]. Approximately 70% of colon cancers in Lynch syndrome arise in the proximal colon [3]. Histologically, Lynch syndrome-related colorectal cancers tend to be mucinous and poorly differentiated with signet ring cells [6–8]. Another striking sign of MSI-H colorectal cancer is a high density of tumor-infiltrating lymphocytes [7]. Despite poor histological differentiation, the biological behavior of MSI-H colorectal carcinoma is less aggressive compared to that of MSS colorectal cancer [5–8].

### MSI Deficiency in Sporadic Colorectal Cancers

MSI-H profile is observed in about 12% of sporadic colorectal cancers. The immunohistochemical finding in MSI-H sporadic tumors is usually simultaneous loss of MLH1 and PMS2. This is caused by MLH1 promoter hypermethylation, which is often associated with a BRAF c.1799T>A (p.V600E) mutation. This somatic mutation turns off the production of MLH1 mRNA and, therefore, the MLH1 protein is absent in cancer cells (Fig. 1) [30, 31]. The mutation of BRAF V600E gene is distinct in sporadic MSI-H tumors and not observed in tumors with germline mutations [32].

If loss of MLH1/PMS2 protein expression is observed, analysis of BRAF V600E mutation or analysis of methylation of the MLH1 promoter should be conducted to rule out a sporadic case. If tumor is MLH1/PMS2-deficient and somatic BRAF mutation is not detected or MLH1 promoter methylation is not identified, this MSI-H is less likely to be sporadic, and testing for germline mutations is indicated (Table 2).

## Prognostic Value of MSI Status for Colorectal Cancers

MSI-H colorectal cancers, including sporadic and Lynch syndrome-related familial colorectal cancers, tend to have a less aggressive clinical behavior and a favorable prognosis compared to MSS tumors [33, 34]. This phenomenon has been repeatedly observed in retrospective studies, large trial studies and meta-analysis [35–38]. For instance, a recent meta-analysis from 32 studies with 1277 MSI-H cases included 7642 patients with stage I–IV colorectal cancers. The authors [38] found that cancers with MSI-H have a significantly better prognosis compared to those with MSS, i.e., intact mismatch repair. The estimated hazard ratio (HR) for overall survival associated with MSI-H was 0.65 (95% confidence interval (CI), 0.59 to 0.71). This benefit was maintained restricting analyses to clinical trial patients (HR = 0.69; 95% CI, 0.56 to 0.85) and patients with locally advanced colorectal cancers (HR = 0.67; 95% CI, 0.58 to 0.78). In a study of 2940 patients with stage I–III colorectal cancers who underwent complete resection, Kim et al. [39] noted that patients with MSI-H had a better clinical prognosis and these tumors were more often associated with local recurrence or peritoneal metastases, while the extra-abdominal recurrence was less frequent compared to MSI-L/MSS tumors. Mohan et al. [40] carried out a single-center study including 1250 colorectal patients and found that MSI-H was associated with a reduced risk of nodal and distant metastases, with an improved disease free survival (DFS) in stage I/II colorectal cancers. However, when MSI-H tumors progressed to stage III, these patients had worse outcomes and the tumors exhibited more aggressive pathological features including higher rates of lymphovascular invasion and perineural invasion than stage I/II MSI-H tumors. In a pooled analysis of 3063 patients from four phase III studies in first-line treatment of metastatic colorectal cancers, Venderbosch et al. [41] found patients with deficient MMR (dMMR) had significantly reduced progression-free survival and overall survival for advanced colorectal cancers and this poor prognosis of dMMR appears to be driven by BRAF mutation. These results suggest that the better prognosis of MSI-H tumors is more apparent for stage I/II early disease, but this predictive trend gradually disappears or even turns into a negative correlation as tumor progresses into advanced stages.

It has been suggested that the improved prognosis of MSI-H cancers may be resulted from the pronounced anti-tumoral immune response of the host [42–44]. Tumors with MSI-H are hypermutated and express abundant peptides that serve as neoantigens to elicit a brisk immune response characterized by abundant tumor-infiltrating lymphocytes, including formation of lymphoid aggregates, medullary growth pattern and Crohn-like lymphocytic reaction [5, 7, 42, 43]. This represents an active immune response to the tumor, a known positive prognostic factor for colorectal carcinoma [42–44]. Due to unstable and hyper-mutational nature, colorectal cancers with MSI-H profile also tend to express high level of checkpoint proteins, including programmed death 1 (PD-1) and programmed death ligand 1 (PD-L1) [45], which makes MSI-H tumors more responsive to immunotherapy with PD-L1/PD-1 blockade (see the following discussion).

## Predictive Value of MSI Status for Colorectal Cancer Treatment

### MSI Status and Chemotherapy Response

Fluorouracil (5-FU) combined with leucovorin is considered as standard care for patients with stage II colorectal cancer. Data indicate that 5-FU-based adjuvant chemotherapy is ineffective in stage II cancer patients with MSI-H [46], consistent with the preclinical data showing that MMR deficiency is associated with 5-FU resistance in colorectal cancer cells [47, 48]. Given the fact of favorable prognosis and lack of benefit from 5-FU-based adjuvant chemotherapy, many authors suggest that stage II colorectal cancer patients with a MSI-H phenotype should not be referred to adjuvant chemotherapy [49–53].

The chemotherapy regimen of folinic acid, 5-FU and oxaliplatin (FOLFOX), which is the standard adjuvant treatment regimen after surgical resection of stage III (lymph node-positive) cancers or for metastatic disease, however, does appear to be effective in patients with MSI-H cancers, similar to their MSS countertype [54–57]. Therefore, stage III colorectal cancer patients can be offered for adjuvant FOLFOX treatment, irrespective of MMR status. However, so far only limited data are available from prospective clinical trials and further studies are required. Nevertheless, knowing the MSI status of the patient may help oncologists to draw up a most appropriate, personalized treatment plan.

### MSI Status and Immunotherapy Response

Recent studies have shown that metastatic colorectal cancers with MSI-H respond favorably to immune checkpoint inhibitors [11, 58, 59]. These tumors tend to have high expression of checkpoint proteins, including PD-1 and PD-L1, which interfere with the body’s antitumor T cell response. By disabling these proteins, checkpoint inhibitors enable T cells to attack and kill tumor cells, allowing the immune system to do its job more effectively [11, 58–60].

Le et al. [11] conducted a phase II trial in patients with metastatic colorectal cancers with or without MMR deficiency to evaluate the clinical activity of an antibody to PD-1 receptor, called pembrolizumab (Keytruda). Patients with MSI-H colorectal cancers and other carcinomas with MSI-H profile, including endometrial, gastric, small bowel carcinomas and ampullary or cholangiocarcinoma, had high rates of immune-related objective response (40 and 71%, respectively) and high rates of immune-related progression-free survival at 20 weeks (78 and 67%). No responses were seen in MSS colorectal cancer patients, and the 20-week progression-free survival was only 11%.

More recently, the promising antitumor activity of nivolumab (Opdivo), another anti-PD-1 monoclonal antibody, in patients with MSI-H metastatic colorectal cancers was also sustained in an update of the phase II CheckMate-142 trial presented at the 2017 Gastrointestinal Cancers Symposium [61]. At a median follow-up of 7.4 months (range, 0.3–25.3), the overall response rate with single-agent nivolumab in a cohort of 74 MSI-H patients was 31.1%. The median progression-free survival (PFS) was 9.6 months (95% CI, 4.3–NE), and the 12-month PFS rate was 48.4% (95% CI, 33.6–61.7). The median overall survival (OS) had not been reached (95% CI, 17.1–NE), and the 12-month OS rate was 73.8% (95% CI, 59.8–83.5). Treatment was well-tolerated, with no new safety signals.

These results suggest that MSI status may have significant implication in therapeutic options. Immune checkpoint blockade inhibition is less toxic than chemotherapeutic regimens and can provide significant benefits to the advanced cancer patients whose conditions are usually weak. If microsatellite stability is validated as a biomarker of immune checkpoint inhibitor efficacy, it will prove exceptionally useful in selecting patients most likely to benefit from such therapies.

In theory, immune checkpoint inhibition may benefit patients with earlier-stage disease as well, and this is an important research question to be addressed in the near future. Positive results in these groups could potentially spare these patients’ chemotherapy and expand the number of patients who could benefit from less toxic immunotherapy.

## Ere of Universal MSI Testing

Lynch syndrome is currently under-recognized, under-diagnosed and under-managed. For example, an estimated 175,000 people have Lynch syndrome in the UK but a staggering 95% of those do not know they have it. This is due to lacking of efficient, systematic testing system [62]. It is vital that people who have Lynch syndrome are identified, so they can take steps to reduce their risk of recurrence or for family members, of preventing cancer from developing.

MSI testing with either immunohistochemistry or PCR-based method is found to be cost-effective, sensitive, specific and is getting widely accepted. Therefore, several organizations including the US Multi-Society Task Force on Colorectal Cancer [26], the US National Comprehensive Cancer Network (NCCN) [63] and the UK National Institute for Health and Care Excellence (NICE) [64] have recommended testing all patients with colorectal cancer for Lynch syndrome; the tumor should be tested for mismatch repair by either DNA analysis or immunohistochemistry for MLH1, MSH2, MSH6 and PMS2 proteins.

Expanding MSI testing to all people with colorectal cancer will certainly increase the detection of Lynch syndrome. This can lead to increased surveillance and improved patient outcomes through earlier diagnosis and treatment. Meanwhile, given the evolving role of MSI in prognostic estimation and therapeutic efficiency prediction, the universal testing will have important implications in colorectal cancer diagnosis and personalized therapeutic approach.

## Conclusion

Screening for MSI-H colorectal cancers is clinically significant in detecting Lynch syndrome, predicting prognosis and determining the application of oncological treatment for colorectal cancers. Recent studies on the potential role of MSI in targeted immunotherapy for metastatic colorectal carcinomas have shown that testing MSI status in these patients may be critical in precision medicine [11, 58–60]. There is a clear trend toward universal testing of newly diagnosed colorectal cancers for evidence of microsatellite instability. Notably, some of extra-colonic cancers, such as endometrial carcinoma, gastric carcinoma and ovarian carcinoma, also exhibit MSI-H profile, and it is possible that MSI status may eventually become an important prognostic and predictive marker in tumors beyond the colon [65–67]. At the present moment, multiple clinical trials are ongoing, and in the near future, our knowledge about MSI in targeted immunotherapy will increase as a consequence of the completion of these ongoing studies.
